# Evaluation and Numerical Investigations of the Cyclic Behavior of Smart Composite Steel–Concrete Shear Wall: Comprehensive Study of Finite Element Model

**DOI:** 10.3390/ma15134496

**Published:** 2022-06-26

**Authors:** Hadee Mohammed Najm, Amer M. Ibrahim, Mohanad Muayad Sabri Sabri, Amer Hassan, Samadhan Morkhade, Nuha S. Mashaan, Moutaz Mustafa A. Eldirderi, Khaled Mohamed Khedher

**Affiliations:** 1 Department of Civil Engineering, Aligarh Muslim University, Aligarh 202002, India; ameralburay@gmail.com; 2 Department of Civil Engineering, College of Engineering, University of Diyala, Diyala 32001, Iraq; 3 Peter the Great St. Petersburg Polytechnic University, 195251 St. Petersburg, Russia; mohanad.m.sabri@gmail.com; 4 Department of Civil Engineering, VPKBIET, Baramati, Pune 413133, India; samadhanmorhade@gmail.com; 5 Faculty of Science and Engineering, School of Civil and Mechanical Engineering, Curtin University, Bentley, WA 6102, Australia; nuhas.mashaan1@curtin.edu.au; 6 Department of Chemical Engineering, College of Engineering, King Khalid University, Abha 61421, Saudi Arabia; maldrdery@kku.edu.sa; 7 Department of Civil Engineering, College of Engineering, King Khalid University, Abha 61421, Saudi Arabia; kkhedher@kku.edu.sa; 8 Department of Civil Engineering, High Institute of Technological Studies, Mrezgua University Campus, Nabeul 8000, Tunisia

**Keywords:** composite steel plate shear wall, hysteresis curves, ductility, energy absorption, finite element model

## Abstract

The composite shear wall has various merits over the traditional reinforced concrete walls. Thus, several experimental studies have been reported in the literature in order to study the seismic behavior of composite shear walls. However, few numerical investigations were found in the previous literature because of difficulties in the interaction behavior of steel and concrete. This study aimed to present a numerical analysis of smart composite shear walls, which use an infilled steel plate and concrete. The study was carried out using the ANSYS software. The mechanical mechanisms between the web plate and concrete were investigated thoroughly. The results obtained from the finite element (FE) analysis show excellent agreement with the experimental test results in terms of the hysteresis curves, failure behavior, ultimate strength, initial stiffness, and ductility. The present numerical investigations were focused on the effects of the gap, thickness of infill steel plate, thickness of the concrete wall, and distance between shear studs on the composite steel plate shear wall (CSPSW) behavior. The results indicate that increasing the gap between steel plate and concrete wall from 0 mm to 40 mm improved the stiffness by 18% as compared to the reference model, which led to delay failures of this model. Expanding the infill steel plate thickness to 12 mm enhanced the stiffness and energy absorption with a ratio of 95% and 58%, respectively. This resulted in a gradual drop in the strength capacity of this model. Meanwhile, increasing concrete wall thickness to 150 mm enhanced the ductility and energy absorption with a ratio of 52% and 32%, respectively, which led to restricting the model and reduced lateral offset. Changing the distance between shear studs from 20% to 25% enhanced the ductility and energy absorption by about 66% and 32%, respectively.

## 1. Introduction

The components of composite steel plate shear walls (CSPSWs) consist of web plates, infill steel plates, concrete, and shear studs. The composite steel plate shear wall proves to be excellent over reinforced concrete walls in terms of ultimate strength, stiffness, ductility, and energy dissipation [1]. 

Previous studies have demonstrated a high interest in using composite steel plate shear walls in buildings and construction [2,3,4]. The appropriate provisions for composite shear walls, such as various seismic behaviors of composite shear walls, are ASCE 7-10 and AISC 341-10 [5,6], which are prepared by allowing the use of CSPSW systems in earthquake zones. Scholars have conducted numerous experimental tests to examine the behavior of composite shear walls in the absence of boundary walls. Nie et al. [7], Mydin [8], Wright [9], and Wang [10] described that composite shear walls not only have high ultimate strength but also have excellent ductile behavior. The local buckling of the web and fracture failure of corners of the wall are the observed failure modes. Zhang et al. [11] and Zhang et al. [12] showed that more channels reduce the ultimate strength and stiffness of the wall but observed improvement in the ductile and energy dissipation behavior. Lastly, to calculate the ultimate strength, initial stiffness has been proposed. However, all the essential variables are not included in the proposed equation, which leads to moderate results. Therefore, this creates a strong demand to perform an exhaustive numerical analysis of composite shear walls [13,14]. 

Several studies have suggested the equation-based FE analysis for composite shear walls. Nguyen et al. [15], Epackachi et al. [16], and Rafiei et al. [17] developed finite element models and checked their accuracy. The parametric analysis of the connector in the wall shows that an increase in the number of connectors could improve the bearing capacity of the steel plate and that a change in the spacing of the connector could affect the failure mode of the steel plate. Wei et al. [18] studied the axial compression performance of composite shear walls. The effect of distance-to-thickness ratios on the failure mode was studied, and a formula to calculate the axial compression of a composite shear wall has been suggested. The higher axial compression ratio of the wall is beneficial to restrain the internal concrete and enhance the compressive strength of the concrete. Thus, the energy dissipation capacity of the composite shear wall is enhanced [19,20]. Increasing the thickness of the steel plate can increase the stiffness and ultimate bearing capacity of the wall, as the hysteretic curve of the wall is plumper [21,22,23,24]. Epackachi et al. [25] simulated shear walls with different aspect ratios. When the aspect ratio was between 0.6 and 3.0, the coupling effect of the moment and shear force was obviously achieved. The specifications [6,26,27,28] define the formula for the shear capacity of composite shear walls. The formulas for the shear and flexural capacity were given, but the formula for estimating the flexural–shear coupling was not supplied [29]. Kantaros et al. [30] reported a comparison of the mechanical properties of different scaffold designs that, however, featured the same porosity and similar dimensions. Compressive strength testing was conducted in three 3D-printed scaffold designs. Moreover, a finite element study was conducted, simulating the compressive strength testing. The results of the compression testing experiment were found to be in good agreement with the computational analysis results. Nedelcu and Cucu [31] studied the buckling modes identification by an FEA of thin-walled members using only GBT cross-sectional deformation modes. The authors presented the latest developments of an original method based on generalized beam theory (GBT) capable of identifying the fundamental deformation modes of global, distortional, or local nature in general buckling modes provided by the shell finite element analysis (FEA) of isotropic thin-walled members.

In summary, the seismic performance of the CSPSW is typically affected by various factors such as the thickness of the infill steel plate, thickness of the concrete wall, distance between the shear studs, ratio of reinforcement, concrete strength, steel yield strength, and layout of the shear stud [32]. Rahai, A. and Hatami, F. [33] investigated the performance of the composite shear wall and established the base for the research on seismic behavior. Although few analyses have suggested the formulas for calculation of lateral stiffness, ductility, and energy dissipation, they were all based on the test results. The predictive effect with other parameters is unknown. In addition, the previous studies mainly focused on the behavior of traditional CSPSW. 

This study’s objective was to examine the behavior of the smart CSPSW under cyclic load and to investigate the consequence of many parameters on this new type of CSPSW.

## 2. Smart Structure Technology

The research on how to maintain the safety of humans inside buildings is an important issue of concern in modern times, as buildings with a low security level pose a threat to human life. Smart structure technology is a modern building and structure control system that notes its own condition, detects impending failure, monitors the damage, and adapts to changing environments [34,35]. The smart technology of composite steel plate shear wall is installed by adding a gap between the steel frame and concrete wall; this gap is provided to improve the performance of CSPSW. The most important benefits of this gap are improvement in stiffness, ductility, and energy absorption of all the systems [36]. 

**Figure 1:**
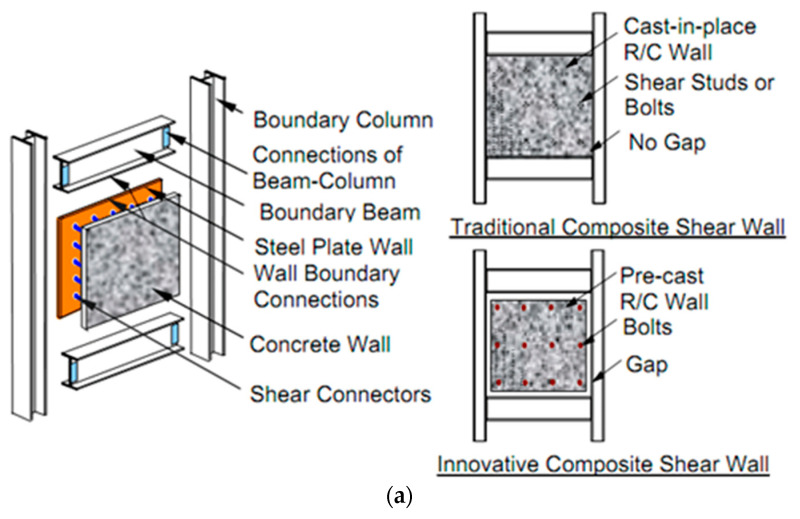

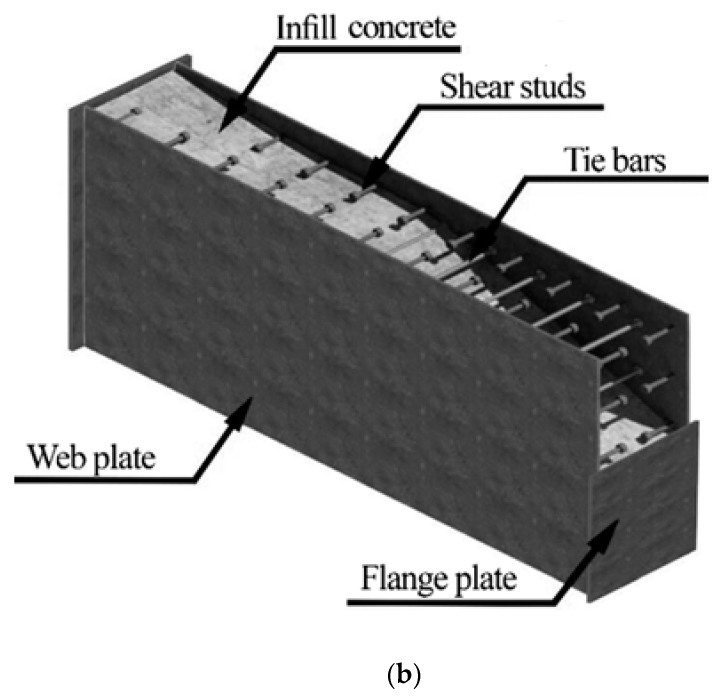
Traditional and innovative composite steel plate shear wall: (**a**) all compounds of the composite shear wall, (**b**) cross-section of composite shear wall.

A smart composite shear wall system consists of an infill steel plate, boundary frame (beam and column), and concrete wall attached on one side of the infill steel plate or both sides (in this research, the steel plate was attached on one side only). The reinforced concrete wall is in mediating contact with the boundary steel frame because there is a gap in between [37]. The difference between the traditional and smart CSPSW is in the change in the behavior of the concrete wall under cyclic loading, whereas in the traditional CSPSW, the concrete wall works in conjunction with a steel plate. However, in smart CSPSW, due to the gap between the steel frame and concrete wall, RC will not work and resist lateral load until the inter-story drift has reached a certain value. Figure 1 shows the structural components of the newly developed shear walls [38,39,40,41].

## 3. Experimental Program

### 3.1. Sample Design

**Figure 2:**
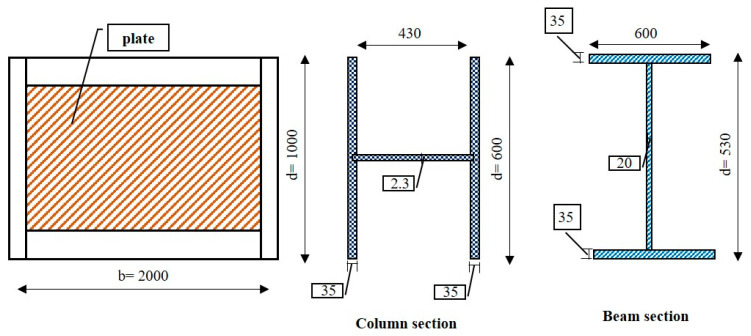
Experimental specimen dimensions.

The experimental work conducted on CSPSW was carried out by Rahai and Hatami [33] to study the behavior of composite shear walls made of concrete and steel. The details of the experimental work of CSPSW are shown in Figure 2.

The frame’s parameters had a length (center to center of column) of 2000 mm and height (center to center of the beam) of 1000 mm. For this model, IPE2000 from ST37 was used for the flexural frame strengthened with 12 mm plates connected to both flanges. Steel of 3 mm thickness was used for the plates. The thickness of the concrete plate was 50 mm, reinforced with 1% of concrete volume.

There was a 30 mm gap between the concrete cover and the boundary elements. In order to connect the concrete cover to a steel plate, 7 mm diameter *100 mm length bolts were used. Moreover, to reinforce the concrete, a 6.5 mm reinforcing bar diameter was used with a center-to-center distance of 65 mm [31]. Table 1 shows the characteristics and strength of steel that was used in all models St37 with yield stress of 240 MPa and ultimate stress of 370 MPa. The steel’s behavior was considered a bilinear elastic–plastic curve for modeling. The compressive strength of concrete in the 28th day’s cylindrical core sample is 45 MPa, and its tensile strength is equivalent to 3 MPa.

### 3.2. Loading Program and Test Setup

**Figure 3:**
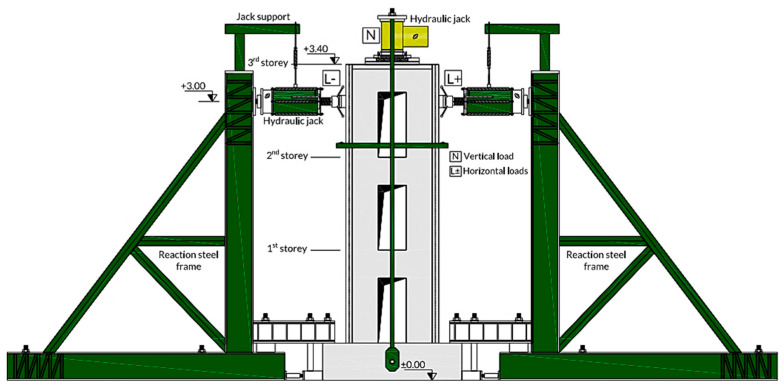
Cyclic loading arrangement.

Horizontal loading was controlled by force [33]. In the force loading phase, the horizontal forces were 600 kN, and loading was cyclically loaded with 1/60 Hz frequency. The experimental load characteristics are shown in Table 2. The loading history is illustrated in Figure 3.

## 4. Finite Element Model (FEM)

### 4.1. Model Overview

#### 4.1.1. Part and Element of the FE Model

The numerical study was performed by finite element analysis using ANSYS. The finite element model consists of five parts: the infilled steel plate, shear studs, outer steel frame, reinforced concrete wall, and reinforcement. The outer steel frame consists of a web and flange plate.

The four nodded shell 181 elements from the ANSYS element library were used to model the outer steel frame, infilled plate, web, and flange plate. The element has six degrees of freedom at each node. The change in stress in the thickness direction cannot be ignored in shear stud and concrete walls because the sizes in the three directions have little difference. Therefore, the element choice to represent the shear stud and reinforcement was a link 180. The element used to represent the concrete wall was 3D solid65. In the test, the shear studs were welded on the web plate. Thus, the shear studs were tied to the steel plate in the FE model. In the test, the reinforcement and the steel studs were fixed in the concrete. 

#### 4.1.2. Contact of FE Mode

**Figure 4:**
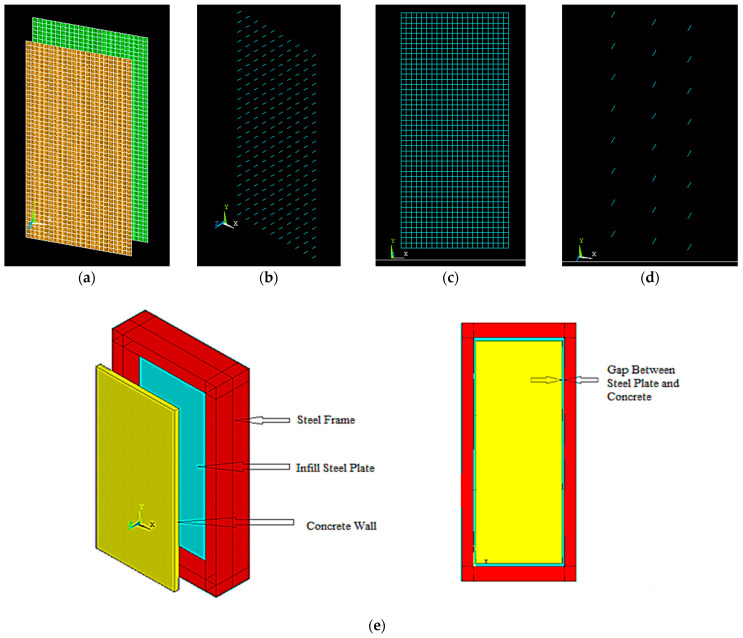
Contact between different elements: (**a**) interface surface; (**b**) load slip; (**c**) reinforcement; (**d**) shear stud; (**e**) assembled FEM.

The friction contact model has been used between the steel plate and concrete. The tangential friction coefficient is 0.6 [37], as shown in Figure 4a. Interface surface between infill steel plate and concrete wall represented by targe170 and contact 174. Finally, the load slip action is represented by comb 39, as shown in Figure 4. The assembled FE model is shown in Figure 4e.

#### 4.1.3. Boundary Conditions

The bottom frame is a fixed-end constraint, and the boundary condition of the top beam is a sliding constraint. Therefore, six degrees of freedom are constrained at the bottom steel frame (i.e., U1 = U2 = U3 = UR1 = UR2 = UR3 = 0), and four degrees of freedom are constrained at the top steel frame (i.e., U3 = UR1 = UR2 = UR3 = 0). 

#### 4.1.4. Steel Constitutive Model

Steel plate is a major element in the composite shear wall. Preferably, this plate is chosen of steel with a low yield point. For example, an St37 steel plate is preferred for high-strength steel plates because an St37 steel plate, due to its low yield point, is preferred to encourage the yielding of steel plates. 

#### 4.1.5. Concrete Constitutive Model

The reinforced concrete cover on one side or both sides of a steel plate carries some of the story shears by improving the diagonal compression field and increasing strength and stiffness. Of course, the major role of the reinforced concrete cover is to prevent out-of-plane buckling of steel plate prior to reaching yielding. In some cases, shear studs not only are subjected to shear but also to a considerable tension due to local buckling of the steel plate. For cast-in-place concrete, welded shear studs are usually utilized; for pre-cast concrete walls, bolts can be used.

### 4.2. Validation of Finite Element Model

**Figure 5:**
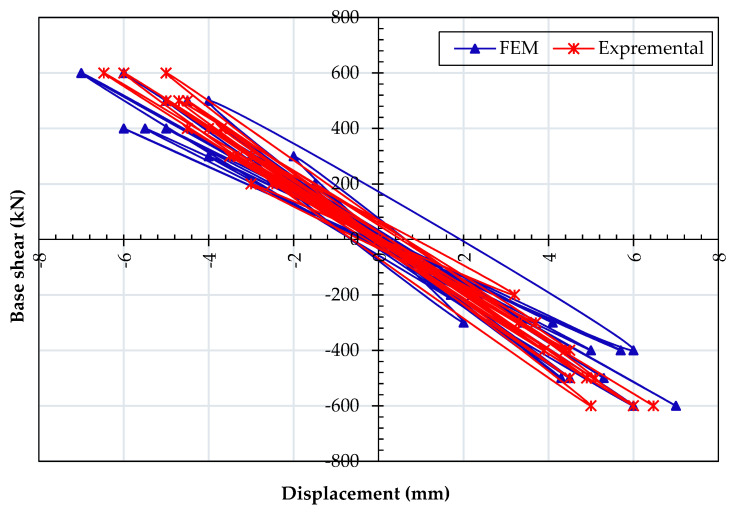
Comparison of deformation load in the numerical and experimental model for CSPSW.

Before performing an actual parametric study, the validation of the FE model was performed. The results of hysteretic curves obtained from FE analysis and test results were compared, as shown in Figure 5. It is observed that predicted FE results are directly matching with actual test results. Therefore, it is concluded that the FE model is able to simulate the hysteretic curves of the composite steel plate shear wall in a significant way.

**Figure 6:**
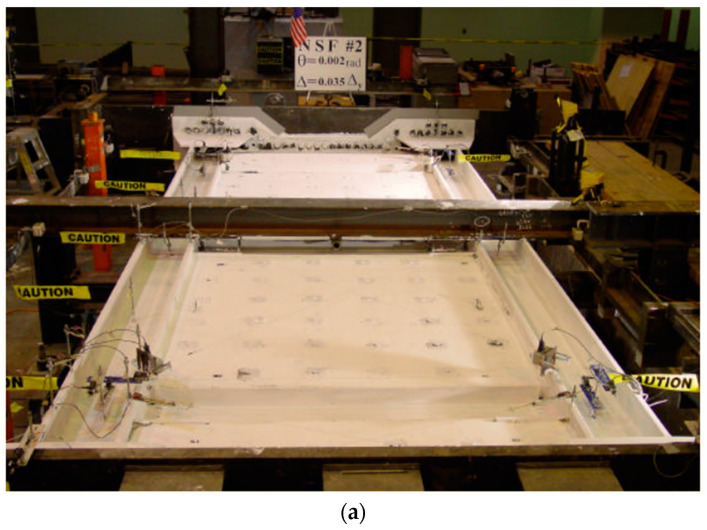

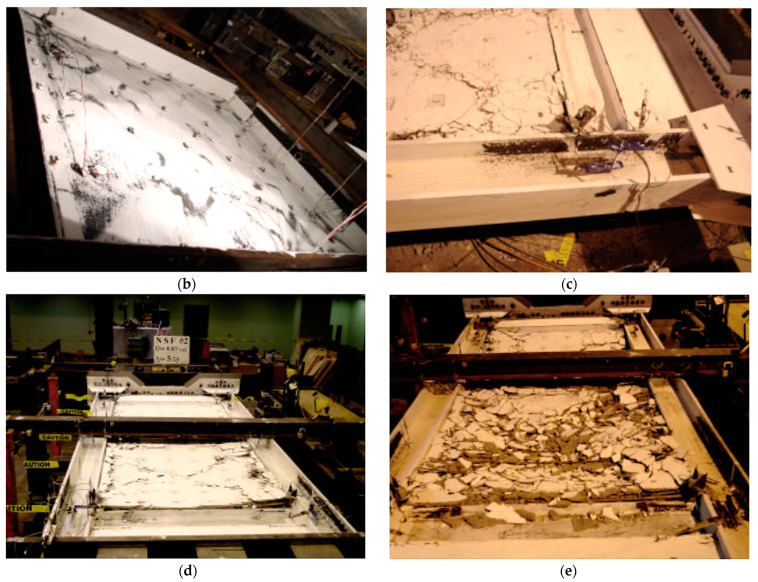
Local buckling, out-of-plane and crack formation for experimental specimen. (**a**) A View of Test Specimen (**b**) steel plate buckling; (**c**) plastic hinge at the base of column; (**d**) concrete crack at mid of the test; (**e**) concrete crack at end of the test.

**Figure 7:**
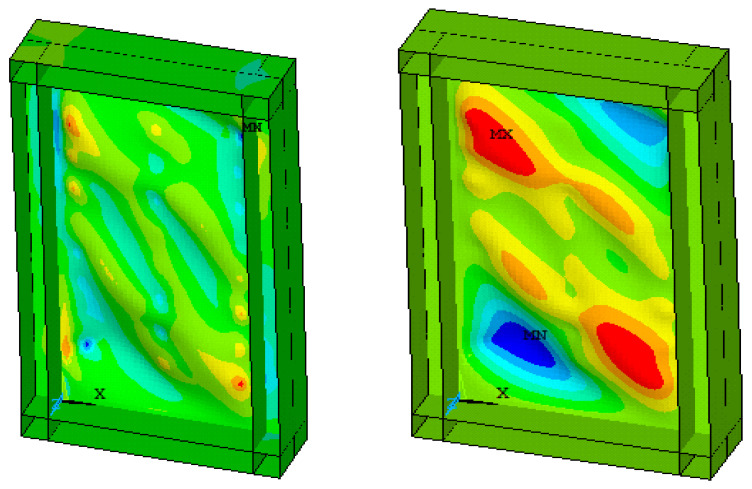
Local buckling, out-of-plane deflection for numerical specimen.

In the test loading process, the steel plate experienced severe buckling at different positions with increasing horizontal displacement as shown in Figure 6. The FE model could simulate the local buckling phenomenon, as shown in Figure 7.

**Figure 8:**
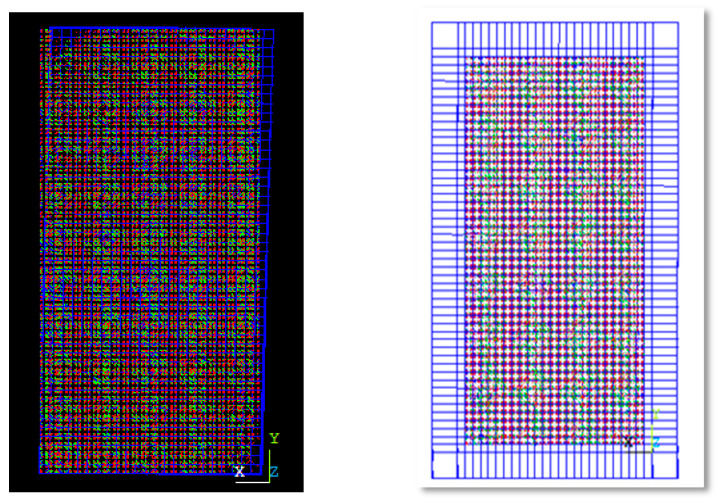
Concrete crack formation for numerical specimen.

The results obtained during the last cyclic loading from the experimental test and the ANSYS output are presented in Figure 6, Figure 7 and Figure 8, which show the comparison of out-of-plane deflection and crack formation in the composite shear wall in different experimental and numerical specimens. The lateral displacement was found to be 6.47 mm and 7 mm in the case of numerical and experimental tests, respectively.

## 5. Parametric Analysis

For optimization of shear wall parameters in tall structures, the parametric study was performed by changing the section size and design guidelines as suggested [33]. The consequences of numerous variables have been vitally studied on the stiffness, ductility, and energy dissipation of the composite shear wall. In order to study the hysteric behavior of the model in parametric analysis, the cyclic load was applied to the wall. The boundary conditions in the model were consistent with the test. 

The variables consisted of the gap between the concrete wall and steel frame, the thickness of the infill steel plate, the concrete wall, the distance between the shear studs, the ratio of reinforcement, concrete strength, steel yield strength, and layout of a shear stud. The selected standard model parameters are different, and many were chosen as follows: the gap between the concrete wall and steel frame was 40 mm, concrete wall thickness was 50 mm, the steel ratio was 1%, and infill steel plate thickness was 3 mm. In addition, a distance of 200 mm between the shear studs was selected, the axial compressive strength of the concrete was 45 MPa, the yield strength of the infill steel plate was 240 MPa, and the layout of the shear stud (H*V) was 3*8.

In the parametric analysis, the gap had a range of 0–30–40–50 mm, the thickness of the infill steel plate had a range of 3–6–12 mm, the thickness of the concrete wall had a range of 50–75–100–150 mm, and the distance between the shear studs had a range of 200–210–220–230–240–250 mm.

### 5.1. Influence Rules of the Parameters

The influence rules of key design parameters are studied by including material displacement, stiffness, ductility, and energy dissipation. The structural behavior of the composite steel plate shear wall, when subjected to cyclic load, is characterized by four different stages when increasing the applied load; these stages are:

- The initial elastic stiffness phase;
- The shear yielding stiffness phase;
- The post-yielding stiffness phase;
- The pre-failure stiffness phase.

Meanwhile, the smart CSPSW, at first loading, displays a linear elastic response where the steel frame and infill steel plate, beams and columns, undergo inelastic deformations. 

After that, the interaction between the infill steel plate and the reinforced concrete panel in the compression field is extra efficient. While the lateral loading is further raised, the infill steel plate responsible is immaterially and geometrically nonlinear. Moreover, the lateral shear stiffness of the wall decreases substantially owing to the shear yield of the infill steel plate. 

During the third phase, with the excess of lateral unloading, the pure shear yield transpires pending the full shear yield appearing in the infill steel plate, and the lateral stiffness reduces gradually at this phase.

While the lateral load surpasses the shear yield capacity of the infill steel plate, the material and geometric nonlinearity responsible for steel frame and boundary elements is massive. At this phase, the frame supplies utmost lateral stiffness.

From the results, it can be seen that increasing the model thickness (infill steel plate and concrete wall) worked to increase the structural strength capacity and the model’s ability to absorb and dissipate energy, which led to a delay in the model failure; at the same time, it prevents the rapid drop in the load-carrying capacity.

Similarly, increasing the distance and layout of the shear studs (certified number of shear studs) increased the structural strength capacity and enhanced the ability of the model to absorb energy and the model ductility.

What is more, if the properties of smart CSPSW were increased, the structural strength capacity and model ability to absorb and dissipate energy would be enhanced.

#### 5.1.1. The First-Group Models (Influence of Gap between Concrete Wall and Steel Frame)

1. Lateral displacement

**Figure 9:**
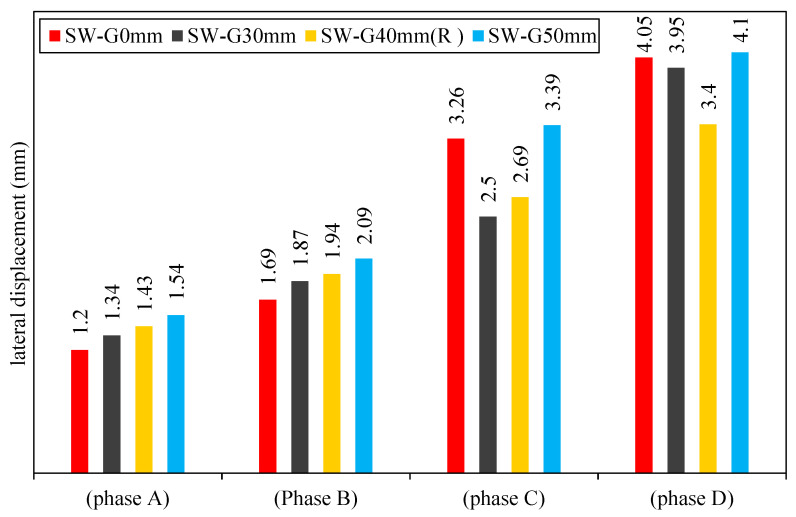
Lateral displacement of group 1.

After loading the first-group models SW-G0mm, SW-G30mm, SW-40MM (R), and SW-G50mm gradually, it can be noted that the model passed through four phases as explained below. Furthermore, lateral displacement of group 1 at each phase can be seen in Figure 9.

- Phase A:

In this phase, the applied load causes linear relation between the horizontal unload and the resulting displacement. This relationship for the models SW-G0mm, SW-G30mm, SW-40MM (R), and SW-G50mm continued until yield deflection was reached.

**Figure 10:**
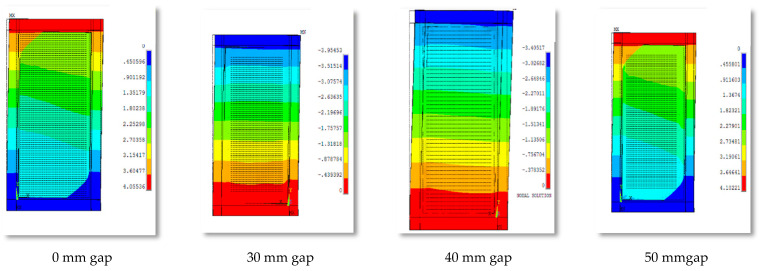
Out-of-plane displacement for the various gaps between the steel frame and concrete wall.

In this phase, the lateral displacement of models SW-G30mm, SW-40mm (R), and SW-G50mm was larger by 12, 17, and 28%, respectively, as compared with the reference model SW-G0mm. By increasing the lateral load, the first yield will occur in the steel plate defined. The out-of-plane displacement of group 1 for 0 mm, 30 mm, 40 mm, and 40 mm gaps is shown in Figure 10. 

It is worth mentioning that, in this stage, all the models of traditional and smart composite steel plates with a gap of 30, 40, and 50 mm, respectively, were symmetric in their behavior until they reached the shear yield zone.

- Phase B:

This phase began at the shear yield zone, which represents the first point of the transformation curve to a flat line that has a high incline, as a result of the high increase in the specimen deflection. 

When increasing the gap between the steel frame and concrete wall, the interaction between the reinforced concrete panel and infill steel plate in the compression zone became more active. Thus, the results caused a decrease in the lateral shear stiffness for this model. The shear yield zone of the models SW-G30mm, SW-40mm (R), and SW-G50mm was found to be larger by 11, 15, and 24% as compared to the reference model SW-G0mm.

- Phase C:

During this phase, when lateral load increases, the shear yield spreads until the full shear yield occurs in the infill steel plate; the models with gaps gave good results under increased lateral load.

For models with a gap of 30 and 40 mm, the lateral displacement was lower than that of the model without a gap of SW-G0mm by 23 and 20%. However, this displacement for models with a gap (50) mm was larger than that of reference model SW-G0mm by 4%.

- Phase D:

This phase refers to the pre-failure of the models. In CSPSW, the infill steel plate of CSPSW resists lateral load by pure shear yield, as a reinforced concrete panel prevents inelastic buckling. 

In this phase and in the same cycle loading (600 KN), the deflection of SW-G50mm is larger by 14% compared with the reference model SW-G0mm. Meanwhile, for the other models (SW-G30mm and SW-G40mm (R)) at the same cycle loading, the deflection was lower by 2% and 16%, respectively.

In this phase, all the models show a nonlinear inelastic reaction of the steel frame, and the lateral stiffness is supplied by steel boundary elements. It is worth mentioning at this stage that the symmetry between the behavior of SW-G50mm and the behavior of SPSW shows the large gap between the steel frame and concrete wall, which leads to the formation of cracks in the concrete before contact between them. Furthermore, the system loses the idea of a smart composite steel plate shear wall, which depends essentially on concrete contribution delay to work with steel frame.

2. Stiffness

**Figure 11:**
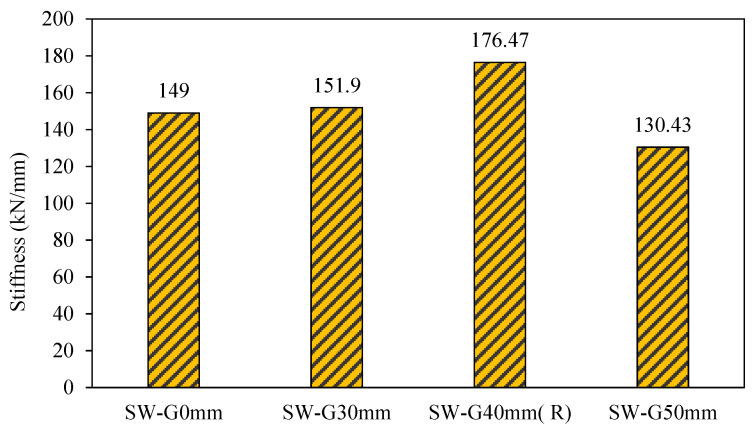
Stiffness of group 1.

Stiffness is the ratio of load vs. deformation and can be used to describe either the elastic or plastic (after yield) range. It can be seen from the load-deflection curve that the stiffness refers to the slope of the curve at any point along the curve. Figure 11 presents the initial elastic stiffness (K_e_) and the proportion of the initial elastic stiffness of SPSW to CSPSW (K_ec_/K_es_) (Rahai and Hatami, 2009).

Figure 11 shows the stiffness for all group one models. From this result, it can be noted that when increasing the gap to 30 and 40 mm for SW-G30mm and SW-40mm (R), the stiffness was increased by 2 and 18%, respectively, in comparison with the reference model SW-G0mm. Consequently, the model resistance deformations increased. However, in the model of gap 50 mm, its stiffness decreased significantly by 13% as compared to the reference model SW-G0mm, where the specimen was resistant to lateral load. SW-40mm (R) has terrific stiffness among all the models of the first group, and stiffness is equal to 176.47 kN/mm.

3. Ductility

Ductility refers to the deformation that a material can undergo after it has yielded or exceeded its elastic range. From the load-deflection curve, it is concluded that ductility refers to the length of the curve after the yield point to failure. The ductility ratio is calculated as the ratio of the maximum displacement to the yield displacement ($\mu =\delta_{\max}/\delta_{y}$). The yield point displacement ($\delta_{y}$) is calculated through the notion of equal plastic energy. Hence, the area bounded by the perfect elastic–plastic curve is equal to that of the actual push-over curve [30] (Shafaei et al., 2016). 

**Figure 12:**
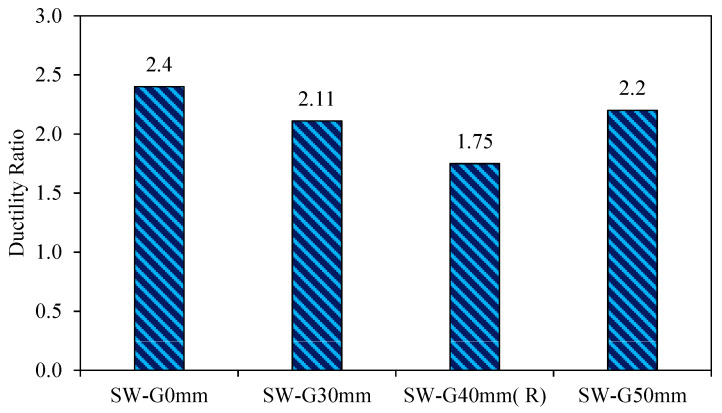
Ductility ratio of group 1.

The results of Figure 12 show that the model SW-G50mm has minimal ductility up to 1.71 as a result of the high value of the deflection at the ultimate load, and this caused sudden buckling and rapid drop in the load-carrying capacity when the ultimate load was achieved.

The great ductility of models with a small gap was because of the high estimation of the lateral displacement at an ultimate load contrasted and the lateral displacement value at the yield load and, in this manner, brought on a gradual failure in the model load capacity.

From the above results, it appears that there was a proportional relationship between the ductility and the gap between the steel frame and concrete wall; therefore, increasing the gap resulted in a decrease in the ductility due to the decreased moment of inertia when the gap was increased. Consequently, this affects the ability of shear wall to resist the lateral load.

4. Energy Absorption

Energy absorption ability is a critical indicator of the model’s resilience to loading. Models with high energy absorption ability are typically found to have high imperviousness to impact and crash loading and hence are valuable for high-performance structures.

The absorbed energy by a shear wall in a half-cycle can be objectively accepted as the zone under shear load displacement, from which the region of recoverable elastic is not subtracted. It is assumed that the unloading and the elastic moduli are approximately the same. For instance, when the max cycle load of the reference model is equal to 600 kN, the displacement value will be about 4.01 mm, and its curve will take a parabola shape. Therefore, energy absorption is equal to (1/3*displacement*load), where the former law represents the area of the parabola shape [30] (Shafaei et al., 2016). 

**Figure 13:**
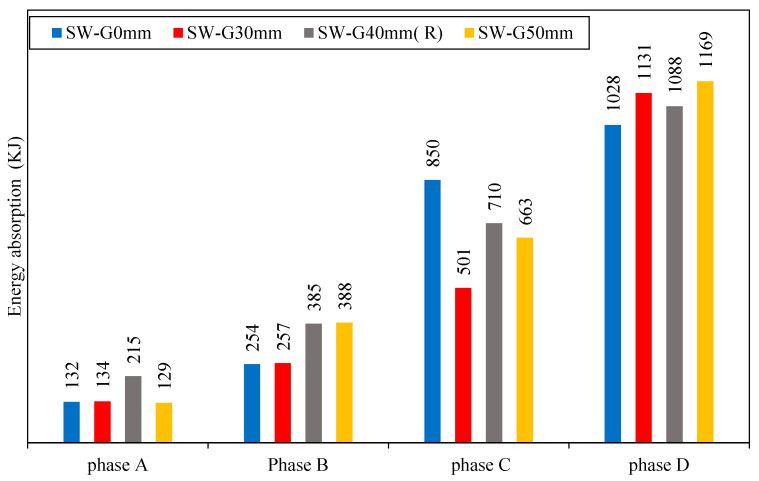
Energy absorption of group 1.

Figure 13 shows the energy absorption of each model through each phase. Through phase B, it can be noticed that an increased gap between the steel frame and concrete wall could increase the energy absorption by 2, 34, and 35% for SW-G30mm, SW-G40mm (R), and SW-G50mm, respectively, as compared to reference model SW-G0mm. 

Models with the gap (SW-G30mm, SW-G40mm (R), and SW-G50mm) had good energy absorption, which was due to the high area under the curve of load deformation, and it referred to the increase in the resistance of the model to the deformation.

In phase D, it can be seen that the energy absorption of the models SW-G30mm, SW-G40mm (R), and SW-G50mm was larger by 10, 6, and 12% as compared to model SW-G0mm.

From the results of the phases above and the calculation of stiffness, ductility, and energy absorption, it is noticed that the gap between the steel frame and concrete wall should be limited by a specific value of 4% of the width because this value gives a good result, which results in a delay in the failures of the model, and this model is economical in the amount of concrete. Therefore, the other groups of smart CSPSW can use SW-G40mm (R) as a reference model.

#### 5.1.2. The Second-Group Models (Influence of Infill Steel Plate Thickness)

1. Lateral displacement

**Figure 14:**
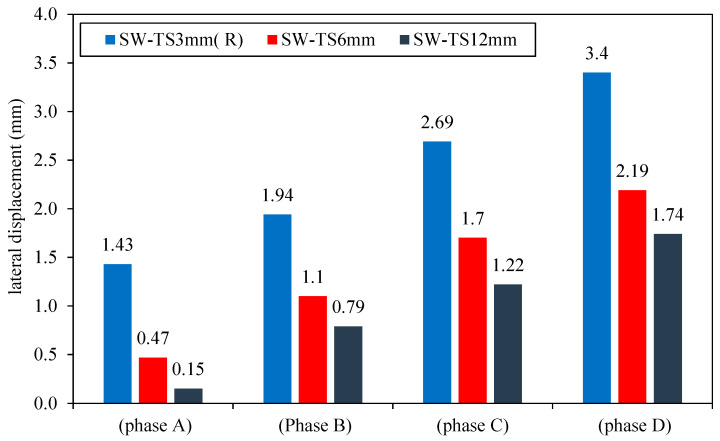
Lateral displacement of group 2.

The second group, models SW-TS6mm and SW-TS12mm, was loading gradually; the lateral displacement of group 2 at each phase can be seen in Figure 14. Moreover, it can be noted that they passed through four phases depending on the applied load, as discussed below.

- Phase A:

The applied loads cause a linear relationship between the lateral load and the resulting displacement. This relationship for models SW-TS6mm and SW-TS12mm continued until yield displacement was achieved. In this stage, the lateral displacement value of the models SW-TS6mm and SW-TS3mm (R) was lower by 67 and 89% as compared with the reference model SW-TS3mm (R), as a result of an increase in the smart CSPSW thickness of infill steel plate which caused a decrease in the yield displacement and increase in the yield load values which led to an increase in the elastic stage for the models SW-TS6mm and SW-TS12mm as compared to the reference model SW-TS3mm (R).

- Phase B:

In this phase, increasing the thickness of the infill steel plate caused an increase in the strain hardening capacity for these models and led to an increase in the stress redistribution significantly until ultimate displacement was achieved. For SW-TS6mm and SW-TS12mm, the lateral displacement was lower by about 43–59% as compared to the reference model SW-TS3mm (R). At the same load, as a result of the increased thickness of the steel plate of smart CSPSW, there was a decrease in the yield displacement and increase in yield load values that led to an increase in the elastic stage for all the models, as compared to reference model SW-TS3mm (R).

- Phase C:

During this phase, when increasing the lateral load, the shear yield propagated until the full shear yield occurred in the infill steel plate, therefore increasing the thickness of the infill steel plate from 3 to 6 and 12 mm. The lateral displacement was lower by 36% and 54%, respectively, as compared to the reference model SW-TS3mm (R).

- Phase D:

The collapse of the reference model SW-TS3mm (R) began before the SW-TS6mm and SW-TS12mm. When comparing the result in this phase at the same cycle loading, it can be noticed that the lateral displacement of SW-TS6mm and SW-TS12mm is lower by 35 and 48%, respectively, as compared with the reference model SW-TS3mm (R). At this stage for SW-TS12MM, the frame provides the most lateral stiffness. Furthermore, by increasing the load, the frame reaches its collapse mechanism, and lateral stiffness declines gradually to a near-zero value.

**Figure 15:**
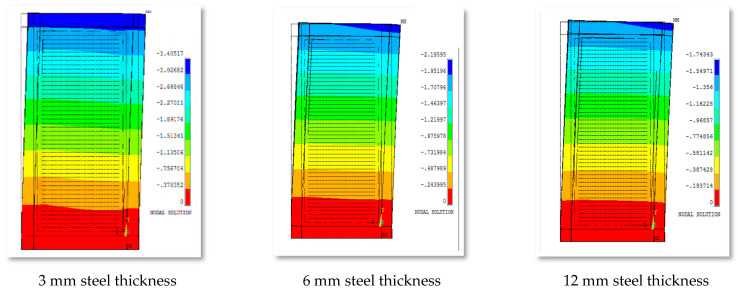
Out-of-plane displacement of group 2 for various steel thicknesses.

Generally, it can be pointed out that the thickness of steel has a substantial effect on the lateral displacement, as shown in Figure 15.

2. Stiffness

**Figure 16:**
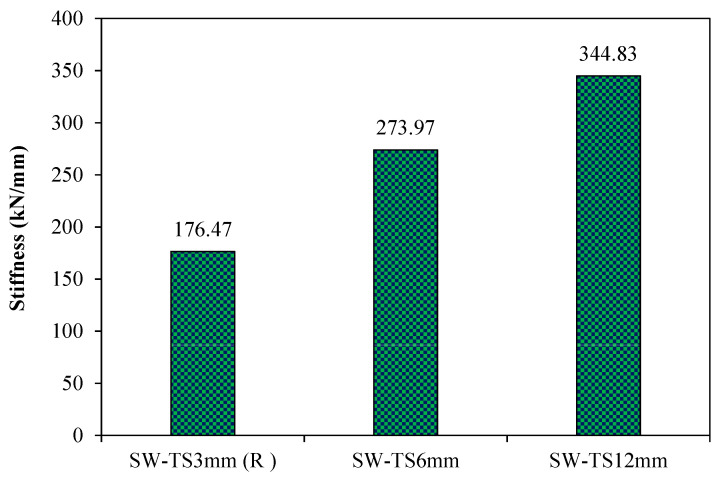
Stiffness of group 2 (thickness of infill steel plate).

Figure 16 demonstrates the stiffness values for the second-group models. From this result, it can be noted that increasing the thickness of the infill steel plates from 3 to 6 and 12 mm led to an increase in their stiffness by about 55% and 95%, respectively, as compared to the reference model SW-TS3mm (R). Thus, it can be concluded that the stiffness of the models was directly compared to the infill steel plate thickness.

3. Ductility

**Figure 17:**
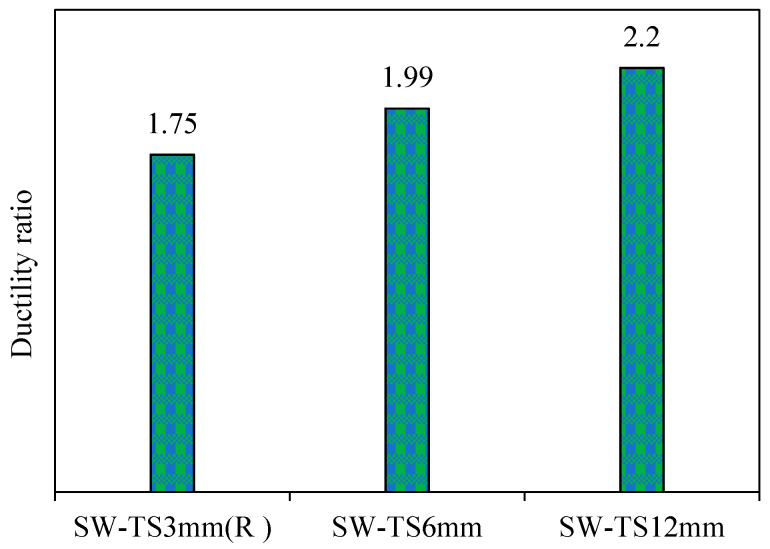
Ductility ratio of group 2.

Figure 17 shows the ductility ratio of all the models when increasing the thickness of the infill steel plate. From Figure 17, it can be found that the models SW-TS6mm and SW-TS12mm have larger ductility, up to 12% and 21%, respectively, as compared to the reference model SW-TS3mm (R). It was because of the high estimation of the deflection at an ultimate load and the deflection value at the yield load, and in this manner, it brought on a continuous failure in the model failure limit.

Therefore, it seems that there is a proportional relationship between the ductility and the infill steel plate thickness; consequently, increasing the thickness causes an increase in the ductility.

4. Energy Absorption

**Figure 18:**
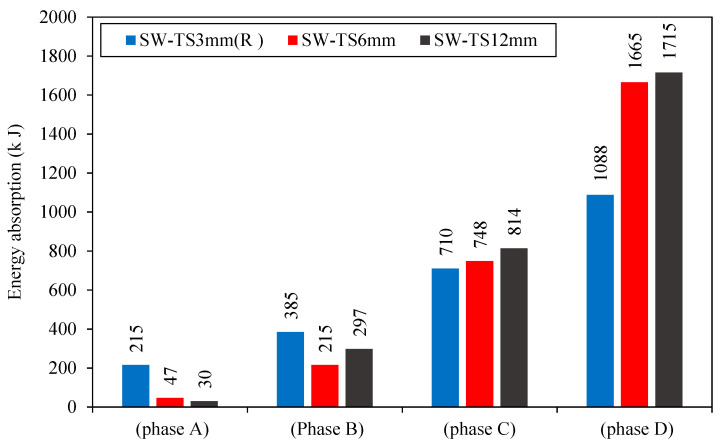
Energy absorption of group 2.

Figure 18 shows the energy absorption of each model through each phase. For SW-TS6mm and SW-TS12mm in phase C, when there was an increase in the thickness of the infill steel plate, the energy absorption increased by 5 and 14% as compared to the reference model SW-TS3mm (R). Meanwhile, through phase D, it is noticed that an increase in the thickness of the infill steel plate resulted in an increase in the energy absorption by 53 and 57% for SW-TS6mm and SW-TS12mm as compared to the reference model SW-TS3mm (R). 

Models with a large thickness (SW-TS12mm) had great energy absorption, and it was due to the high area under the curve of load deflection. It refers to the increased resistance of the model to the deformation. From the results of the phases above and calculation of stiffness, ductility, and energy absorption, it can be noticed that the thickness of infill steel plate for (2000*1000) mm (length*width) specimen dimensions should be limited by a specific value (min 3 mm) due to the trail thickness of 1 mm of the infill steel plate which results in a quick failure in the model. The type of failure was expressed as an opening in the steel plate. As a result, the best range for using the thickness of the infill steel plate was between 3 and 12 mm. The best value in terms of cost economy was 6 mm.

#### 5.1.3. The Third-Group Models (Influence of Concrete Wall Thickness)

1. Lateral displacement

**Figure 19:**
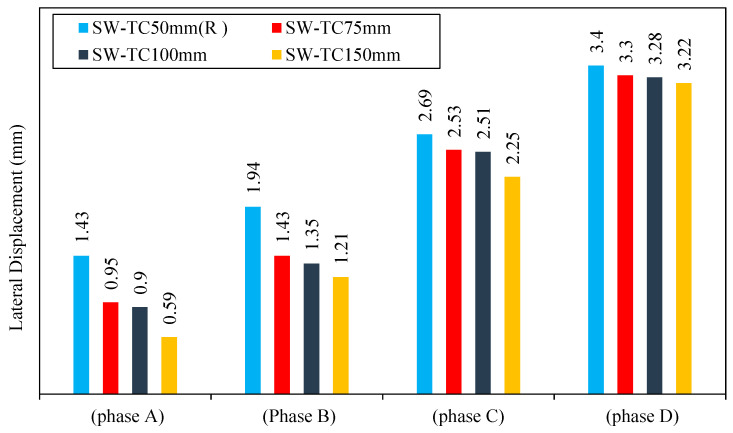
Lateral displacement of group 3.

The third-group models SW-TC75mm, SW-TC100mm, and SW-TC150MM were loading gradually. From the result, it is noted that they passed through four phases depending on the applied load, and the lateral displacement of each phase is shown in Figure 19:

- Phase A:

The applied load causes a linear relationship between the lateral load and the resulting lateral displacement. This relationship for the models SW-TC75mm, SW-TC100mm, and SW-TC150mm continued until yield displacement was achieved. In this phase, the lateral displacement value of the models SW-TC75mm, SW-TC100mm, and SW-TC150mm was lower by 33, 37, and 58% as compared with the reference model SW-TC50mm (R). Increasing the smart CSPSW thickness of the concrete wall caused a decrease in the yield displacement and increase in yield load values and thus an increase in the elastic stage for the specimens SW-TC75mm, SW-TC100mm, and SW-TC150mm as compared to the reference model SW-TC50mm (R).

- Phase B:

After increasing the thickness of the concrete wall, a comparison of the result was performed, and it was noticed that lateral displacement of the models SW-TC75mm, SW-TC100mm, and SW-TC150mm at the same load was lower than the reference model SW-TS3mm (R) by 26, 30, and 37%. This is because of increasing the thickness of the concrete wall of this model, which increases the strain hardening capacity for the models, and that led to an increase in the stress redistribution significantly until data achieved the ultimate displacement.

- Phase C:

When increasing the lateral load, the shear yield propagates until the full shear yield occurs in the infill steel plate. In this phase, after comparison of the result, when increasing the thickness of concrete wall from 50 to 75, 100, and 150 mm, the lateral displacement was lower by 5, 6, and 16%, respectively, as compared to the reference model SW-TC50mm (R).

- Phase D:

The collapse of the reference model SW-TS3mm (R) began before the SW-TC75mm, SW-TC100mm, and SW-TC150mm because of the increased thickness of the concrete wall. Thus, the failure possibility of this model under lateral load was lower than under other loads. Consequently, when comparing the result in this phase at the same cycle of loading, it can be noticed that the lateral displacement of SW-TC75mm, SW-TC100mm, and SW-TC150mm is lower by 3, 4, and 5%, respectively.

**Figure 20:**
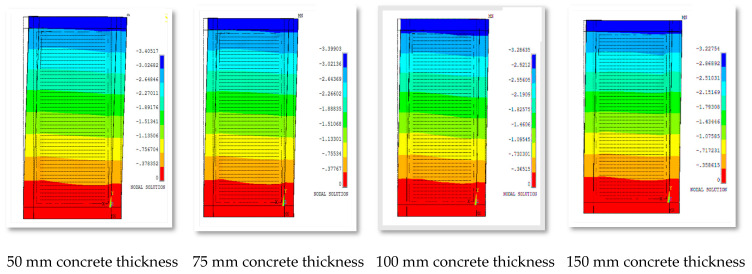
Out-of-plane displacement of group 3 for different concrete thicknesses.

In general, it was observed that the thickness of concrete has an influence on the term lateral displacement, as shown in Figure 20.

2. Stiffness

**Figure 21:**
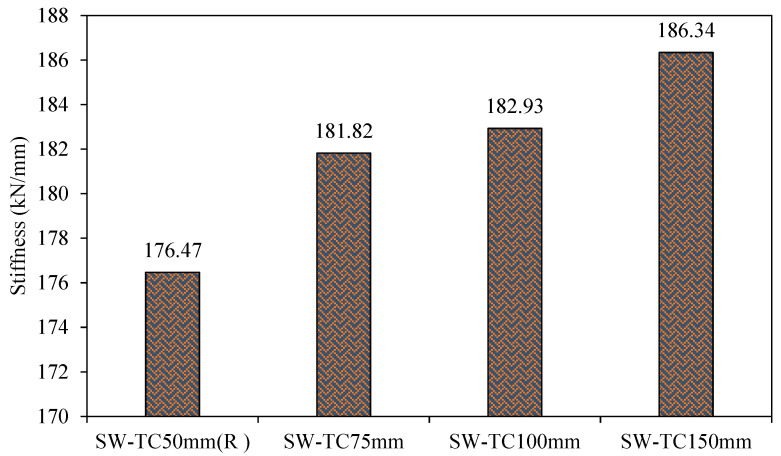
Stiffness of group 3.

Figure 21 shows the stiffness values for third-group models. From these results, it can be concluded that increasing the thickness of concrete wall from 50 to 75, 100, and 150 mm leads to an increase in its stiffness by 3, 4, and 5%, respectively, as compared to the reference model SW-TC50mm (R). Thus, it can be noted that the thickness of concrete wall has a slight effect on the model’s stiffness.

3. Ductility

**Figure 22:**
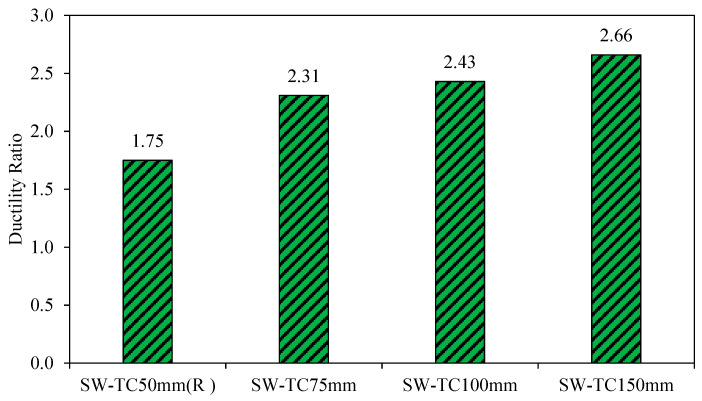
Ductility ratio of group 3.

The values of ductility of the third-group models when increasing the thickness of the concrete walls are shown in Figure 22. After comparison of the result, it can be found that the models (SW-TC75mm, SW-TC100mm, and SW-TC150mm) have large ductility of 32, 38, and 52%, respectively, as compared to the reference model SW-TC50mm (R). This result was due to a high value of lateral displacement at the ultimate load compared to the deflection value at the yield load and thus caused a gradual failure in the model load capacity.

Thus, it appears that there is a proportional relationship between the ductility and the concrete wall thickness; therefore, increasing the thickness causes an increase in the ductility and ultimately gives a gradual drop in the load-carrying capacity until the failure load is reached.

4. Energy Absorption

**Figure 23:**
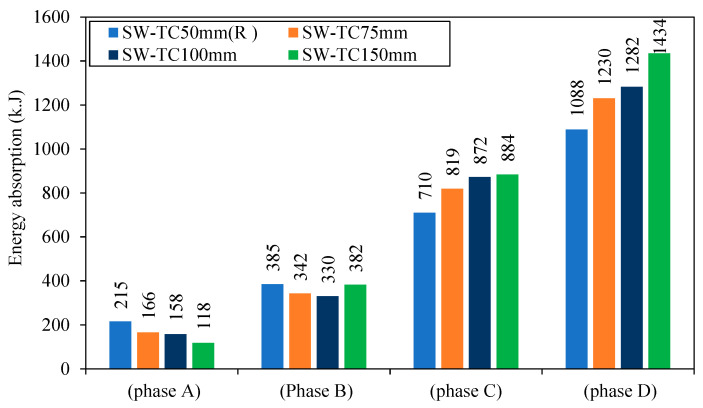
Energy absorption of group 3.

The energy absorption of each model through each phase is shown in Figure 23. For SW-TC75mm, SW-TC100mm, and SW-TC150mm in phase C, when there is an increase in the thickness of the concrete wall, the energy absorption increases by 15, 23, and 24%, respectively, as compared to reference model SW-TC50mm (R).

Meanwhile, through phase D, it can be noticed that an increased thickness of concrete wall increased the energy absorption by 13, 18, and 32%, respectively, for SW-TC75 mm, SW-TC100 mm, and SW-TC150 mm as compared with reference model SW-TC50mm (R). The models with the large thickness (SW-TC150mm) had good energy absorption, and it was due to the high area under the curve of load deformation. It refers to the increased resistance of the model to the deformation.

From the calculation of stiffness, ductility, and energy absorption, it can be noticed that concrete wall has a large effect on the behavior of smart CSPSW because increasing the thickness of concrete wall leads to an increase in the contribution of concrete in force transfer; therefore, the influence of lateral load on the infill steel plate becomes low. Moreover, increased thickness leads to restricting the frame and reducing the lateral offset. 

From the results of the phases above, the thickness of concrete wall for (2000*1000) mm (length*width) specimen dimensions should be limited by a specific value (max 150 mm) because the behavior of smart CSPSW remains the same beyond that thickness. Therefore, the best range for using the thickness of the concrete wall was 50–100 mm.

#### 5.1.4. The Fourth-Group Models (Influence of Distance between Shear Studs)

1. Lateral displacement

**Figure 24:**
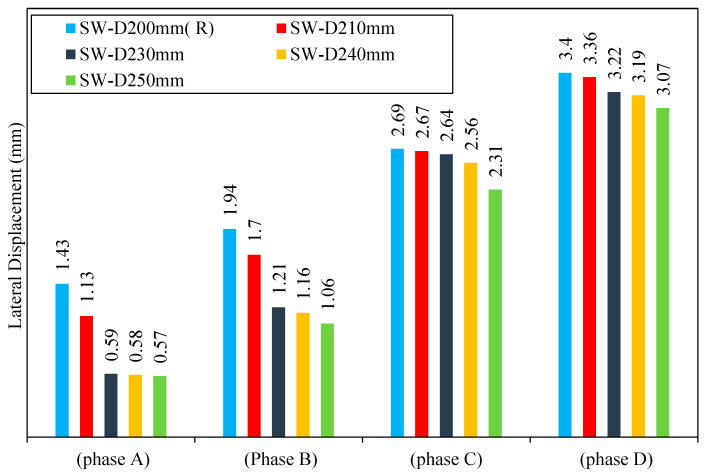
Lateral displacement of group 4.

From Figure 24, it can be seen that the models SW-D210mm, SW-D220mm, SW-D230mm, SW-D240mm, and SW-D250mm go through the same phases as the reference model SW-D200mm (R) when loaded gradually, which are as follows:

- Phase A:

For the models SW-D210mm, SW-D220mm, SW-D230mm, SW-D240mm, and SW-D250mm, the elastic phase starts from the beginning of loading to the yield displacement. The lateral displacement values of the models SW-D210mm, SW-D220mm, SW-D230mm, SW-D240mm, and SW-D250mm were lower by 20, 30, 58, 59, and 60% as compared to the reference model SW-D200mm (R). At the same load, decreasing the distance between the shear studs of smart CSPSW, which causes a decrease in the yield displacement and increase in the yield load values, leads to an increase in the elastic stage for all the models as compared to the reference model SW-D200mm (R). Therefore, it can be noted that a decreased distance between shear studs in all the models has a large effect on the elastic stage for these models.

- Phase B:

The strength capacity of the models SW-D210mm, SW-D220mm, SW-D230mm, SW-D240mm, and SW-D250mm was governed by the plastic deformation, which occurred because of the moment capacity and the shear force at the shear studs. This moment capacity of the models decreases due to a decrease in distance between shear studs (increased number of shear studs) because of the occurrence of a high reduction in the moment contribution of these models. Consequently, the lateral displacement of the models SW-D210mm, SW-D220mm, SW-D230mm, SW-D240mm, and SW-D250mm was lower by 12, 24, 37, 40, and 45% as compared to the reference model SW-D200mm (R). From this, it can be noted that the presence of increased distance between shear studs affected significantly the shear yield phase through the escalation of strain hardening capacity and led to a significant change in the escalation models’ stress redistribution compared with the reference model SW-D200mm (R).

- Phase C:

In this phase, all models of the fourth group had very similar behaviors with very close values of the yield load and yield displacement. It is also observed that the increased distance between shear studs in the CSPSW models did not have a large effect on the post shear yielding phase for these models. In this phase, when there was a variation in the distance between shear studs of 200–250 mm for SW-D210mm, SW-D220mm, SW-D230mm, SW-D240mm, and SW-D250mm, the lateral displacement was lower by 0.74, 0.74, 1.86, 4, and 14% as compared to the reference model SW-D200mm (R).

- Phase D:

This phase began when the model reached the ultimate load by exposure of all model elements that are situated above and below the shear stud to high stresses. The collapse of the reference model SW-D200mm (R) began with the occurrence of buckling in the infill steel plate because of using many shear studs. In other words, the small distance between the shear studs leads to high plastification around shear studs because of exposure to high compression, which leads to a global buckling failure mode.

After the comparison of the result in this phase at the same cycle of loading, it is noticed that the lateral displacement of SW-D210mm, SW-D220mm, SW-D230mm, SW-D240mm, and SW-D250mm is lower by 1, 2, 5, 6, and 9% as compared to the reference model SW-D200mm (R).

**Figure 25:**
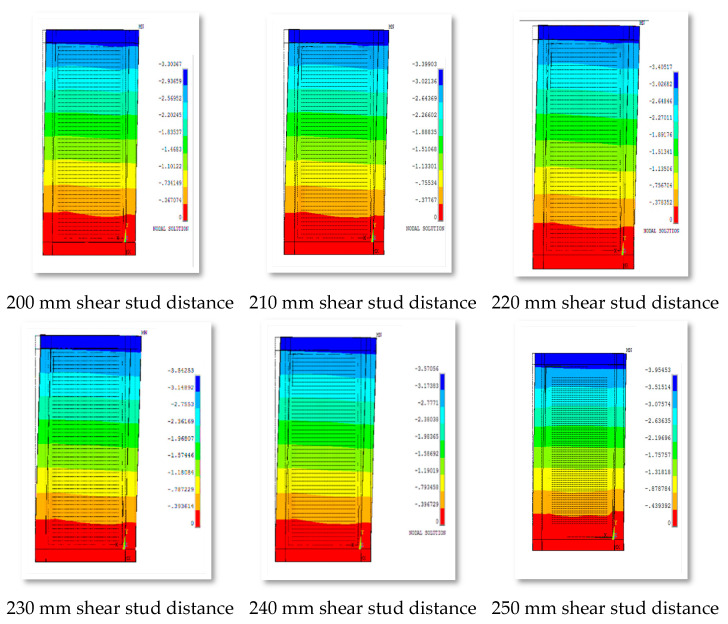
Out-of-plane displacement of group 4 for various shear stud distances.

Overall, it was observed that the distance of the shear stud has a slight effect on the term of lateral displacement, as shown in Figure 25.

2. Stiffness

**Figure 26:**
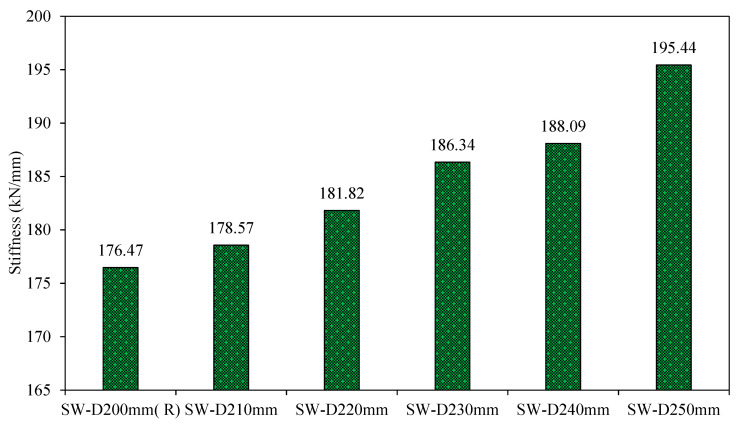
Stiffness of group 4.

Figure 26 gives the values of the stiffness for the fourth-group models. Figure 26 shows that increased distance between shear studs in the models SW-D210mm, SW-D220mm, SW-D230mm, SW-D240mm, and SW-D250mm enhances their stiffness by 1%, 3%, 5%, 6%, and 10%, respectively, compared to the reference model SW-D200mm as a result of the small lateral displacement of these models. From the results, it can be seen that the distance between shear studs has very little effect on the model stiffness.

3. Ductility

**Figure 27:**
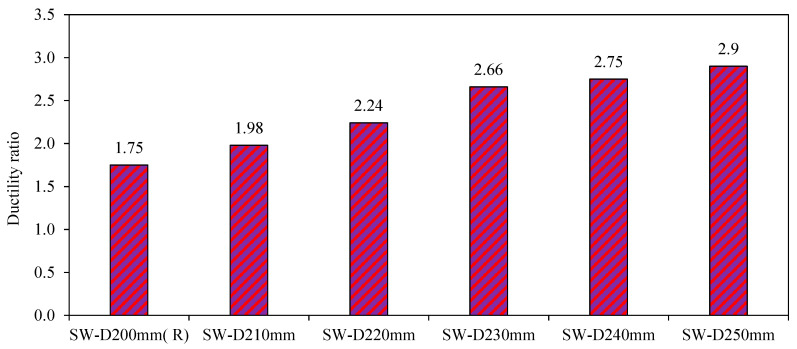
Ductility ratio of group 4.

Figure 27 shows the ductility values of the fourth-group models SW-D210mm, SW-D220mm, SW-D230mm, SW-D240mm, and SW-D250mm. From Figure 27, it can be concluded that the increased distance between shear studs in the models SW-D210mm, SW-D220mm, SW-D230mm, SW-D240mm, and SW-D250mm enhanced their ductility substantially by 12%, 22%, 34%, 37%, and 40%, respectively.

A gradual drop in the load-carrying capacity of these models was observed when they reached the ultimate load compared with the sudden and rapid drop of the reference model SW-D200mm (R).

4. Energy Absorption

**Figure 28:**
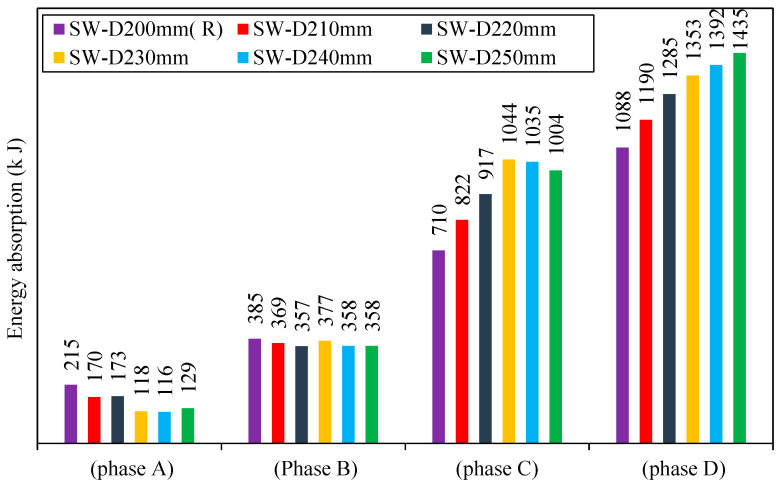
Energy absorption of group 4.

Figure 28 shows the energy absorption of each model through each phase. For SW-D210mm, SW-D220mm, SW-D230mm, SW-D240mm, and SW-D250mm in phase C, when increasing the distance between shear studs, the energy absorption increases by 14, 23, 32, 33, and 30% as compared to reference model SW-D200mm (R). While through phase D, it can be seen that increased distance between shear studs increased the energy absorption by 9, 15, 20, 22, and 24% for SW-D210mm, SW-D220mm, SW-D230mm, SW-D240mm, and SW-D250mm as compared to reference model SW-D200mm (R). The models with a large distance (SW-D250mm) had good energy absorption, and it was due to the high area under the curve of load deflection. This refers to the improved resistance of the model to the deformation. 

From the results of stiffness, ductility, and energy absorption, it is noticed that the distance between shear studs for (2000*1000) mm (length*width) specimen dimensions should be limited by a specific value (250 mm). This is because large distances will cause widespread buckling of the steel plate in free sub-panels between the shear stud and thus will result in no improvement. Therefore, the ideal range for the distance between the shear studs was 200–250 mm.

## 6. Conclusions

Based on the numerical results conducted in this study, the conclusions were drawn as follows:

- Increasing the gap between the steel frame and concrete wall influences the sequences of the yielding of components, where yielding shows in the beam and infill steel plate first. At the end of the test, the columns showed yielding at the base but did not buckle. The gap between the steel frame and the concrete wall should be limited by a specific value of 4% of the width, as this value has a considerable effect on delaying failures of the model. Moreover, this model is economical in terms of the volume of concrete.
- The thickness of infill steel plate for 2000*1000 mm (length*width) specimen dimensions should be limited by a specific value (min 3 mm) because using 1 mm of infill steel plate resulted in a quick failure in the model; the type of failure was expressed as an opening in the steel plate. Therefore, the ideal range of infill steel plate thickness was 3–12 mm. The best value in terms of cost economy is 6 mm.
- The thickness of the concrete wall for (2000*1000) mm (length*width) specimen dimensions should be limited by a specific value (max 150 mm) because the behavior of smart CSPSW remains the same beyond that thickness. Therefore, the best range for using the thickness of the concrete wall was 50–100 mm.
- The distance between shear studs for 2000*1000 mm (length*width) specimen dimensions should be limited by a specific value of 250 mm because large distances will cause widespread buckling of the steel plate and will result in no enhancement. Therefore, the best range for the distance between the shear stud was 200–250 mm.

## Figures and Tables

**Table 1:** Details of the experimental specimens and their mechanical properties.

Group No.	Group Name	SW	Gap	Thickness of Steel	Thickness of Concrete	Distance between Shear Studs	Ratio of Reinforcement	Compressive Strength	Yield Strength	Layout of Shear Stud (H*V)
1	Gap between steel frame and concrete wall	SW-G0mm	0	3	50	200	1%	45	240	3*8
		SW-G30mm	30	3	50	200	1%	45	240	3*8
		SW-G40mm(R)	40	3	50	200	1%	45	240	3*8
		SW-G50mm	50	3	50	200	1%	45	240	3*8
2	Thickness of infill steel plate	SW-TS3mm(R)	40	3	50	200	1%	45	240	3*8
		SW-TS6mm	40	6	50	200	1%	45	240	3*8
		SW-TS12mm	40	12	50	200	1%	45	240	3*8
3	Thickness of concrete wall	SW-TC50mm(R)	40	3	50	200	1%	45	240	3*8
		SW-TC75mm	40	3	75	200	1%	45	240	3*8
		SW-TC100mm	40	3	100	200	1%	45	240	3*8
		SW-TC150mm	40	3	150	200	1%	45	240	3*8
4	Distance between shear studs	SW-D200mm(R)	40	3	50	200	1%	45	240	3*8
		SW-D210mm	40	3	50	210	1%	45	240	3*8
		SW-D220mm	40	3	50	220	1%	45	240	3*8
		SW-D230mm	40	3	50	230	1%	45	240	3*8
		SW-D240mm	40	3	50	240	1%	45	240	3*8
		SW-D250mm	40	3	50	250	1%	45	240	3*8

**Table 2:** Cyclic loading time history.

Time (s)		Max. Load (KN)	Loading Shape	Frequencies (Hz.)
Start	End			
0.0	71	0.0	Cyclic	0.0
72	180	300	Cyclic	1/60
181	360	500	Cyclic	1/60
361	540	600	Cyclic	1/60

## Data Availability

Not applicable.
